# Improving the Understanding of Pathogenesis of Human Papillomavirus 16 via Mapping Protein-Protein Interaction Network

**DOI:** 10.1155/2015/890381

**Published:** 2015-04-15

**Authors:** Yongcheng Dong, Qifan Kuang, Xu Dai, Rong Li, Yiming Wu, Weijia Leng, Yizhou Li, Menglong Li

**Affiliations:** ^1^College of Life Sciences, Sichuan University, Chengdu 610064, China; ^2^College of Chemistry, Sichuan University, Chengdu 610064, China; ^3^College of Computer Science, Sichuan University, Chengdu 610064, China

## Abstract

The human papillomavirus 16 (HPV16) has high risk to lead various cancers and afflictions, especially, the cervical cancer. Therefore, investigating the pathogenesis of HPV16 is very important for public health. Protein-protein interaction (PPI) network between HPV16 and human was used as a measure to improve our understanding of its pathogenesis. By adopting sequence and topological features, a support vector machine (SVM) model was built to predict new interactions between HPV16 and human proteins. All interactions were comprehensively investigated and analyzed. The analysis indicated that HPV16 enlarged its scope of influence by interacting with human proteins as much as possible. These interactions alter a broad array of cell cycle progression. Furthermore, not only was HPV16 highly prone to interact with hub proteins and bottleneck proteins, but also it could effectively affect a breadth of signaling pathways. In addition, we found that the HPV16 evolved into high carcinogenicity on the condition that its own reproduction had been ensured. Meanwhile, this work will contribute to providing potential new targets for antiviral therapeutics and help experimental research in the future.

## 1. Introduction

Human papillomavirus (HPV) has been tantamount to cervical cancer which ranked as the third most common cancer and the fourth most common cause of cancer death, but its actual footprint is much bigger [1, 2]. Persistent infection with mucosal HPV types, especially with HPV16, can also lead to the form of penile, vulvar, vaginal, anal, and oropharyngeal cancer, recurrent respiratory papillomatosis, and certain head afflictions [3, 4]. Furthermore, some data show that the actual number of cases of anal and oropharyngeal cancers is increasing and may have already exceeded (or will soon exceed) that of cervical cancer. HPVs were divided into five different genera: Alpha, Beta, Gamma, Mu, and Nu [5, 6]. HPVs were also classified as cutaneous or mucosal according to their tropism. There are both cutaneous and mucosal HPV for Alphapapillomavirus. Other genera are cutaneous. In addition, 12 mucosal HPVs (HPV16, 18, 31, 33, 35, 39, 45, 51, 52, 56, 58, and 59) were classified as high-risk (HR) HPV types by the International Agency for Research on Cancer (IARC) in 2009 [7, 8]. More than 96.6% of cervical cancer is caused by HR HPVs, while about 54.4% is caused by HPV16. In all HPV-positive noncervical cancers, HPV16 is also the most common HPV type detected. The HPV16 encodes eight proteins: E1, E2, E4, E5, E6, E7, L1, and L2 [9, 10]. These proteins are classified as adaptive proteins which have high carcinogenicity (E5, E6, and E7) and core set (E1, E2, L2, and L1). The E4 protein is embedded within the E2 protein [11].

HPV16 appears to be extraordinary: how can such a small amount of proteins do so much [12]? Protein-protein interaction (PPI) network is a feasible strategy to improve our understanding of its pathogenesis. Several human-pathogen interaction networks have been reported, such as* Plasmodium falciparum*,* Yersinia pestis*, hepatitis C virus (HCV), and Epstein-Barr virus (EBV) [13–16]. Dyer et al. integrated and compared publicly available human-pathogen PPIs from 190 different pathogens to provide a global view of pathogenesis strategies [17]. Unfortunately, it is very limited that PPI pairs between HPV16 and human are obtained by experiment. Therefore, computational methods to predict PPIs have an important role [18]. The SVM with 217-dimensional vector was employed to predict the interactions of HPV16 and HPV18 proteins with human proteins by Cui et al. at the same time [19]. But it is easy to lead overfitting for small sample. In this paper, a new method was employed to represent protein sequence. A support vector machine (SVM) model with sequence and topological features was built to predict new interactions between HPV16 and human proteins. Subsequently, all interactions were filtered and further analyzed by some strategies.

## 2. Methods

### 2.1. Data Sources

We collected human PPIs from large-scale high-throughput screens [20–22] and several interaction databases [23–26], which contained 193,801 interactions among 13,306 proteins. The Pathway Interaction Database (PID) is a growing collection of human signaling and regulatory pathways curated from peer-reviewed literature [27]. As a source of reliable information we extracted about 224 different pathways from the PID. Then the interactions between HPV16 and human proteins were extracted from IntAct [28], APID [29], and VirHostNet [30]. After removing redundancy, a total of 174 interactions were identified and used as positive training set (see Table S1 in the supplementary material available online at http://dx.doi.org/10.1155/2015/890381).

We collected 254 new nonredundant interaction pairs from the literature (see Table S2 in the supplementary material). Finally, the 254 interaction pairs were used as positive test set. It should be noted that whether it was positive training set or positive test set, the interactions were centered on E6 protein and E7 protein because of experimental biases.

### 2.2. Choosing of Negative Set

As a 2-class classification, both positive set and negative set are needed [31]. Positive set is interacting pairs and negative set is noninteracting pairs. Unfortunately, the noninteracting pairs were not readily available. In the absence of negative set, the following strategy was adopted to choose negative set. This strategy was based on such an assumption that proteins locating different subcellular localizations do not interact [32]. First, the all human proteins of human PPI network were grouped into eight subsets based on the eight main types of localization—cytoplasm, nucleus, mitochondrion, endoplasmic reticulum, golgi apparatus, peroxisome, cytoplasm&nucleus and secreted. Then we totaled subsets of human proteins which were targeted by a kind of HPV16 protein denoted as *h*. Therefore, other proteins that did not appear in those subsets were made as candidates who did not interact with *h*. Finally, the same amounts of proteins with targeted human proteins of *h* were randomly picked as negative set of *h*. For example, eight human proteins targeted by E5 protein occupied cytoplasm subset and nucleus subset in positive training set; thus, other human proteins which did not appear in those two subsets were made as candidates and eight proteins of candidates were randomly picked as negative training set of E5 protein.

### 2.3. Feature Extraction

The sequence compositions of the protein pair and the topological features of corresponding human protein were employed to represent protein interaction between HPV16 and human.

In accordance with Shen et al. [33], a protein sequence was represented by three consecutive amino acids. On account of limited sample, however, another class of amino acids was used to reduce the dimension of the vector space of feature vectors. Based on the chemical nature of the side chain of the amino acid, twenty amino acids were classified into five categories: {GAVLIMP}, {STCNQ}, {KRH}, {ED}, and {FYW}. The third category and the fourth category were incorporated into one category, and four categories were considered in total. So there are 4 × 4 × 4 = 64 possible amino acid combinations. The frequency of a combination *k* in a protein *i* was defined as *f*_*ik*_ = *n*_*ik*_/∑_*l*=1_^64^ *n*_*il*_, where *n*_*ik*_ was the occurrences of combination *k* in protein *i*. An interaction between a HPV protein *i* and a human protein *j* was represented by their frequency difference, *d*_*ij*_ = *f*_*i*_ − *f*_*j*_. The parameter *d*_*ij*_ was normalized by

(1) nijk =2×dijk−min⁡dij1,dij2,…,dij64max⁡dij1,dij2,…,dij64−min⁡dij1,dij2,…,dij64  −1,

where *d*_*ijk*_ is the frequency difference of the *k*th combination. The numerical value of *n*_*ijk*_ ranges from −1 to 1.

Besides the standardized frequency difference, degree and betweenness of the human proteins were also used as features. Ultimately, a 66-dimensional vector was built to represent each protein pair. Each interaction was labeled +1 and noninteraction was labeled −1.

The classification model for predicting PPIs was based on support vector machine (SVM) using LIBSVM [34] with the radial basis function (RBF).

There are three differences between our representation and that of Cui et al. [19]. First, twenty amino acids were classified into six classes by Cui et al.: {IVLM}, {FYW}, {HKR}, {DE}, {QNTP}, and {ACGS}. So there are 6 × 6 × 6 = 216 possible amino acid combinations. Second, standardization was done by

(2) $$\begin{array}{c} d_{i}=\left\{e^{\left(f_{i}-\min\left\{f_{1},f_{2},\ldots ,f_{216}\right\}\right)/\left(\max\left\{f_{1},f_{2},\ldots ,f_{216}\right\}-\min\left\{f_{1},f_{2},\ldots ,f_{216}\right\}\right)}\right\}-1. \end{array}$$

Third, a feature element was used to represent the types of virus proteins and was included in a feature vector.

### 2.4. Tissue Specificity Filtering

To ensure utmost biological relevance, tissue specificity filtering was adopted. It has been known that HPVs infect epithelial cells in oral mucosa or skin [6]. In addition, HPVs also lead to recurrent respiratory papillomatosis, head afflictions, and cancers of the cervix uteri, vulva, anus, and oropharynx (including base of the tongue and tonsils) and interact with basal cell and the immune system [3, 35]. We extracted proteins in those cells, tissues, and systems from HPRD [26]. Finally, interactions were filtered by selecting interaction pairs which only contain those proteins.

### 2.5. Enrichment and Pathway Participation Coefficient

The two parameters have been described by Wuchty et al. [13, 36] in detail. But for the sake of completeness, we would describe the two parameters in brief.

Proteins were grouped according to their degree in integrated human PPI network. Each group where each protein has at least *k* interactions was represented by *N*_≥*k*_. In each group the number of human proteins that were targeted by HPV16, *N*_*t*,≥*k*_, was calculated. As a null hypothesis, we randomly sampled protein set from the integrated human PPI network and then calculated corresponding number of targeted proteins, *N*_*t*,≥*k*_^*r*^. Finally the enrichment of targeted proteins was defined as *E*_*t*,≥*k*_ = *N*_*t*,≥*k*_/*N*_*t*,≥*k*_^*r*^. In addition to degree, the same calculation was performed for betweenness. It was noted that *E* > 1 points to an enrichment and vice versa.

For each protein *i* that was involved in pathways and the integrated human PPI network, the corresponding pathway participation coefficient (PPC) in the total set of pathways *P* was defined as PPC_*i*_ = ∑_*p*∈*P*_[|Γ(*i*) ∈ *p*/∑_*p*∈*P*_Γ(*i*)∈*p*|]^2^, where Γ(*i*) ∈ *p* was the set of interaction partners of *i* in the pathway *p*. If a protein predominantly interacted with partners that were members of the same pathway, PPC tended toward 1. Otherwise PPC tended to 0.

### 2.6. GO Term Enrichment

The Gene Ontology (GO) is a hierarchically organized, controlled vocabulary to consistently describe and annotate gene products [37]. GO term enrichment was performed using the DAVID Functional Annotation Chart tool [38, 39]. GO terms are controlled vocabularies that form a directed acyclic graph (DAG), whereby individual terms are represented as nodes connected to more specific nodes by directed edges, such that each term is a more specific child of one or more parents. Therefore, to avoid very general and uninformative GO terms, only GO level 5 terms were considered. The *P* values were corrected for multiple testing using the Bonferroni procedure and transformed by taking the −log⁡_10_⁡ for easier visualization [40, 41].

## 3. Results and Discussion

**Figure 1:**
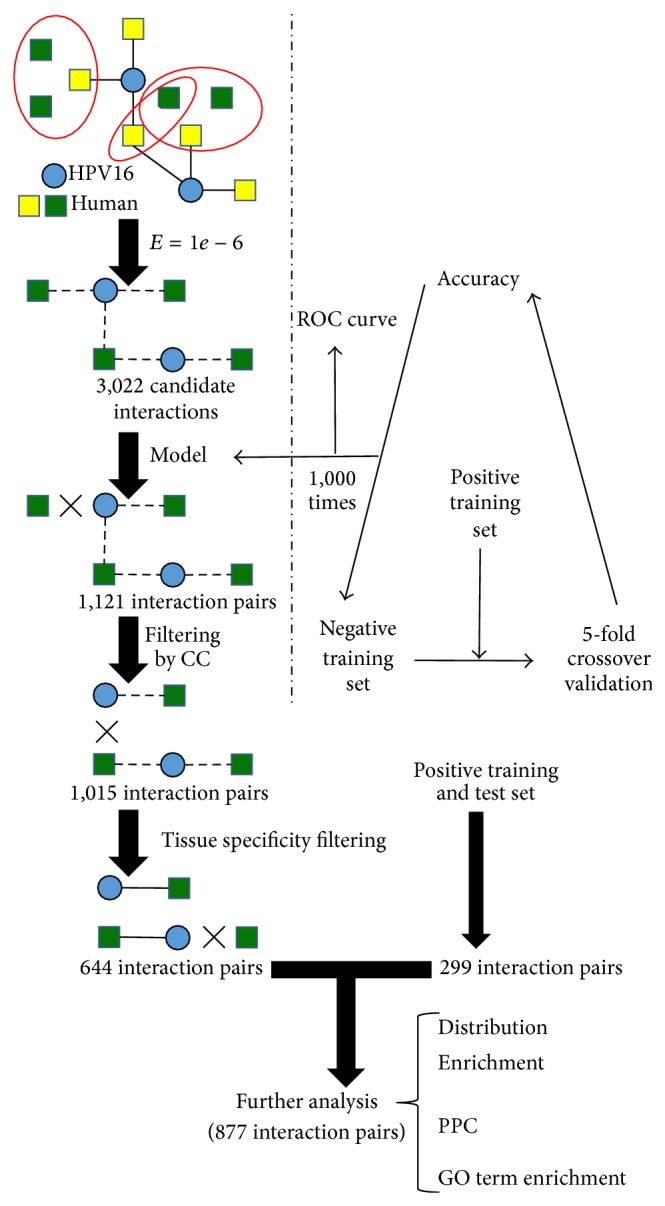
Flowchart to integrate and analyze PPI network between HPV16 and human proteins. A candidate interaction was found, if the human protein had homologs in the human PPI network. This method provided 3,022 candidate interactions. An SVM model was employed to evaluate candidate interactions and 1,121 interactions were left. Subsequently, these interactions were filtered if human proteins with targeted human proteins had the same as cellular component. 1,015 interactions were obtained; positive training set and test set were further filtered by tissue specificity. Finally, 877 interactions were obtained and analyzed. Solid lines delineate validated interactions between virus and human proteins, and dotted lines delineate candidate interactions which would be validated. Homologous proteins are surrounded by ellipse.

We extracted 174 interactions between HPV16 and human proteins and integrated a human PPI network including 193,801 interactions. A flowchart of the whole experiment is shown in Figure 1.

### 3.1. Choosing of Negative Training Set and Evaluating of Model

**Figure 2:**
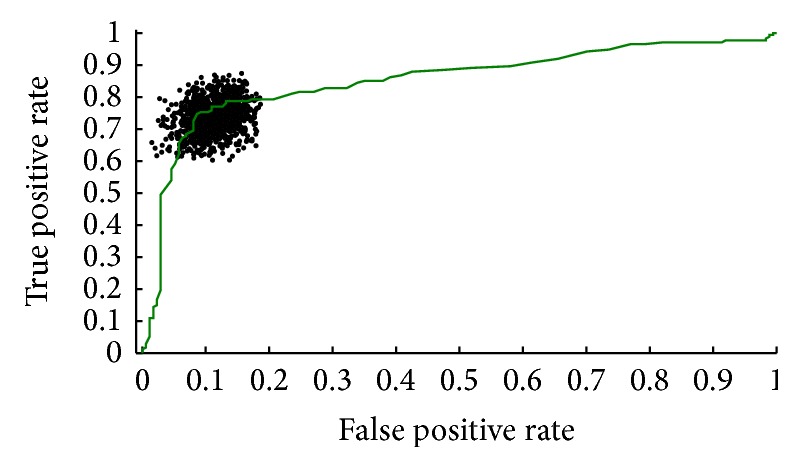
ROC curve of training set. Negative training sets were repeatedly chosen 1,000 times. Applying SVM with 5-fold cross-validation the training sets allowed for a true positive rate TPR = 74.71%, false positive rate FPR = 8.62%, and area under the curve AUC = 0.8627.

The 174 interactions between virus and human proteins were used as positive training set. The selection of negative training set was fundamental to the reliability of the prediction model [33]. Based on a rational assumption, the negative training set was chosen (see Methods section). The SVM with 5-fold cross-validation was employed to optimize the parameters and check the reliability of randomly selected negative training set. Repeating such random trials 1,000 times and calculating average accuracy (81.3 ± 1.3%), we chose a result approaching average accuracy to build model and plot ROC curve (Figure 2) which allowed for a true positive rate TPR = 74.71%, a false positive rate FPR = 8.62%, and area under the curve AUC = 0.8627. Other results were dotted clouds. It was demonstrated that the method of choosing negative training set was significantly reliable and robust.

To evaluate expansibility of the model, a positive test set was collected. Negative test set was selected by the same method with choosing negative training set. Repeating trials 1,000 times, this model, on average, achieved an accuracy AC = 80.0 ± 1.8%, TPR = 78.7%, and FPR = 18.2 ± 3.6%. For comparison, we tested the method of Cui et al. on whole modeling and evaluating. Our method outperformed the method of Cui et al., which, on average, achieved AC = 57.25 ± 1.5%, TPR = 63.4%, and FPR = 47.9 ± 3.1%.

### 3.2. Inferring and Filtering of Candidate Interactions

To find candidate interactions, we ran BLAST with the known targeted human proteins as query sequences against the human proteins in integrated human PPI network. Specifically, we considered a pair of proteins with homology if their *E*-value was <10^−6^. A candidate interaction was detected between a HPV16 protein and homologous protein of targeted human protein. The final set contained 3022 candidate interactions between 8 virus and 1,950 human proteins.

The model built by SVM was applied to evaluate candidate interactions. The 1,121 interactions between 8 virus and 701 human proteins were finally obtained. The 701 human proteins were refined further by selecting human proteins that have the same GO cellular component terms with homologous human proteins from the positive training set. 1,015 interactions were obtained by this refinement. To ensure utmost biological relevance for the 1,015 interactions, tissue specificity filtering was adopted (see Methods section). Filtering interactions provided 644 interactions between 8 HPV16 proteins and 405 human proteins. For simplicity of reference, the filtering result was named as predicted set. Meanwhile, positive set including training set and test set was also filtered by tissue specificity. Finally, all filtering results were combined, providing a total of 877 interactions between 8 virus and 603 human proteins. This set was called as all set.

### 3.3. Distribution of Targeted Human Proteins Based on Host-Virus Interaction

**Figure 3:**
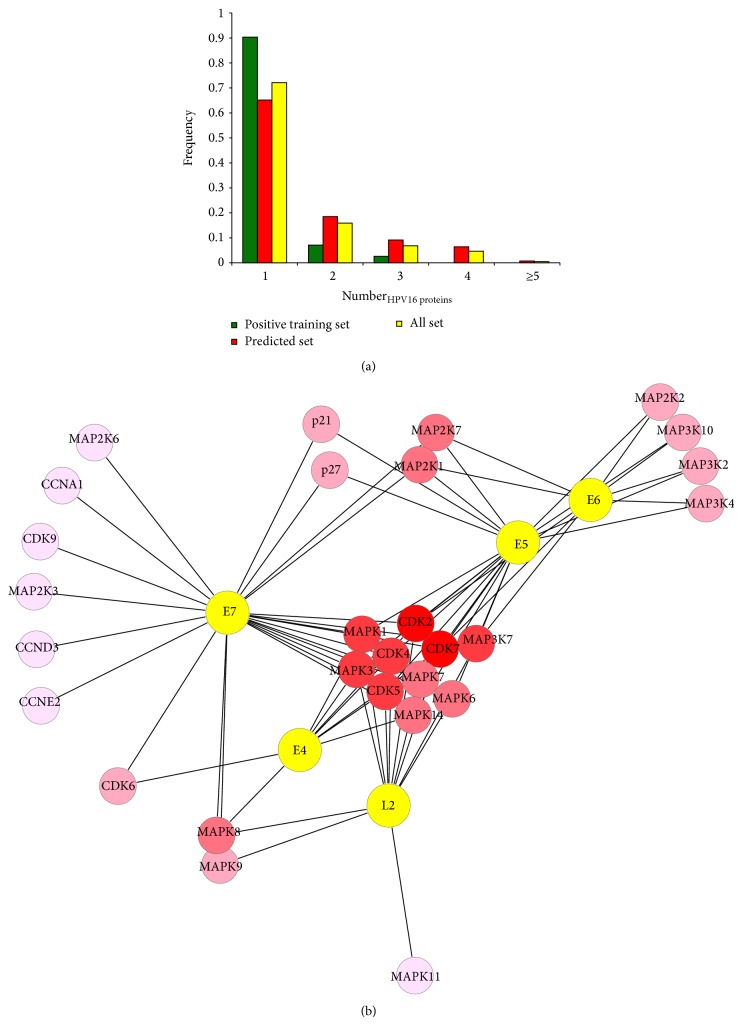
Characteristics of PPI network between HPV16 and human. (a) Whether it was training positive set, predicted set, or all set, a majority of host proteins interacted with a small amount of virus proteins. (b) A network between five virus proteins and some human proteins about cell cycle and phosphorylation cascade. The more virus proteins human protein is targeted by, the darker the node color is.

Now we paid more attention to the all set. The frequency of human proteins that interacted with the same number of viral proteins was calculated. We observed that most human proteins (69.52%) merely interacted with a virus protein in Figure 3(a). The positive training set and the predicted set were addressed by the same calculation method, and their results illustrated similar trend with all set. It suggested that HPV16 interacted with human proteins as much as possible to enlarge its scope of influence by its limited proteins. In order to provide all necessary cellular proteins required for viral replication, the virus has to keep its host cell in cycle [42]. At the molecular level, virus proteins interact with many key cell cycle regulatory proteins, including cyclin-dependent kinase (CDK), cyclin-dependent kinase inhibitors, and cyclin proteins (Figure 3(b)). Among them, CDK2 and CDK7 are the most prominent. The two proteins simultaneously interact with five virus proteins in all set and the five virus proteins are L2, E4, E5, E6, and E7. Combination of CDK2 and some cyclins regulates G_1_/S transition. CDK7 is both a CDK-activating kinase (CAK), which is able to phosphorylate and activate CDK1, CDK2, CDK4, and CDK6 within the activation segment (T-loop) [43–46], and an essential component of the transcription factor TFIIH, which phosphorylates the C-terminal domain (CTD) at Ser 5 of the largest subunit of Pol II [47–49]. These interactions, together with other proteins that bind to HPV16, alter a broad array of cell cycle progression; for example, they block cellular proliferation by causing cell cycle arrest in S-phase [12, 50, 51]. The myosin light chain kinase (MLCK) is also targeted by five virus proteins. It has been proven that MLCK plays a role in the regulation of epithelial cell survival [52] and modulates hypotonicity-induced Ca^2+^ entry and Cl^−^ channel activity in human cervical cancer cells [53]. In addition, HPV16 may be similar to arrest defective-1 that controls tumor cell behavior by MLCK [54].

### 3.4. Statistical Implications of Targeted Host Proteins Based on Human PPI Network

**Figure 4:**
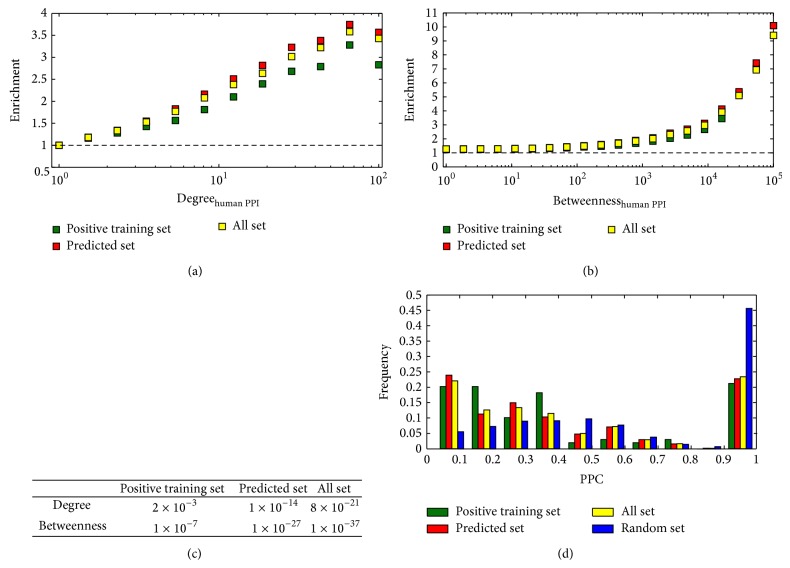
Characteristics of targeted human proteins. (a) The enrichment of targeted human proteins as a function of their degree was calculated. It indicated that hub proteins appeared to be primarily targeted. (b) Analogously, HPV16 tended to interact with bottleneck proteins. (c) *P* values of Fisher's exact tests indicated that HPV16 is highly prone to interact with hub proteins and bottleneck proteins. (d) Considering all set, most proteins have low pathway participation coefficients, which indicated that HPV16 reached into a breadth of signaling pathways. Such a result was shown by positive training set and predicted set.

We calculated the enrichment of targeted human proteins as a function of the degree of human proteins (see Methods section). With an average over 1,000 randomizations, we observed that whether it was all set, predicted set, or positive training set, HPV16 preferred to interact with hub proteins (proteins interacting with a large number of partners) in the integrated human PPI network (Figure 4(a)). Subsequently, we calculated the enrichment of targeted proteins as a function of the betweenness and consistent trend has shown that bottleneck proteins (proteins that are central to many paths in the network) were more affected by virus (Figure 4(b)). Testing the significance that HPV16 tended to interact with hub and bottleneck proteins, we used Fisher's exact test, allowing us to find a statistically significant tendency that HPV16 is indeed highly prone to interact with hub proteins and bottleneck proteins (Figure 4(c)).

We speculated that virus interacted with human proteins as much as possible while tending to influence many signaling pathways to mediate the infection. PPC was adopted to measure this tendency (see Methods section). Focusing on the positive training set, we observed that most human proteins occurred in a variety of pathways through its interaction partners in integrated human PPI network (Figure 4(d)). The predicted set and the all set showed more enforced maxima around low values of PPC. As a comparison, we randomly selected a subset of equal size with human proteins in all set from integrated human PPI network and repeated 1,000 times to calculate average value of PPC. Ignoring the last bar, we found that the random set obeyed the normal distribution, but the all set was linear relationship. Such results strongly indicated that the HPV16 effectively affected a breadth of signaling pathways [13, 55, 56].

### 3.5. Functional Analysis of Targeted Host Proteins

GO term enrichment was employed to perform the comprehensive functional analysis for human proteins of the all set. The main advantage of this approach is that we can make use of term-term relationships, in which joint terms may contain unique biological meaning for a given study [57].

**Figure 5:**
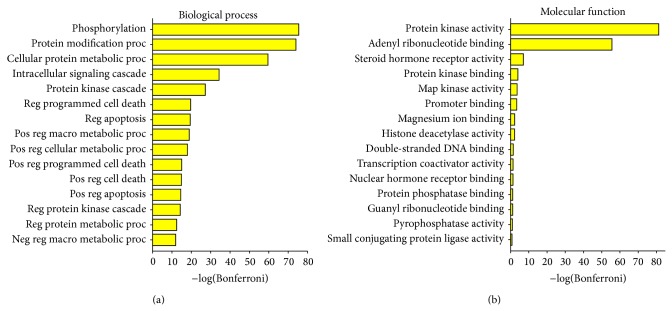
GO term enrichment of all targeted human proteins. (a) Enriched GO biological process terms. (b) Enriched GO molecular function terms. Here only fifteen most significant terms are shown. Bonferroni collected *P* values were transformed by −log⁡_10_⁡. The following abbreviations are used: “reg” is “regulation of,” “pos” is “positive,” “neg” is “negative,” “proc” is “process,” “macro” is “macromolecule,” and “bsyn” is “biosynthetic.”

For all targeted human proteins, significant enrichment was observed in the processes of phosphorylation, metabolism, signaling, cell death and apoptosis, gene expression, and positive or negative regulation terms (Figure 5(a)). This observation was also reflected on the functions which include kinase activity, receptor activity, promoter, DNA binding, and so on (Figure 5(b)). MAPK is a particularly important component in protein kinase phosphorylation cascade. It can enter the nucleus and phosphorylate serine/threonine residues of substrate proteins which contain transcription factors of regulating the cell cycle and cell differentiation. Notably, viral proteins strongly interacted with members of the MAPK family (MAPK1, 3, 6, 7, 8, 9, 11, and 14). Besides MAPK family, partial members of MAP2K and MAP3K family were also targeted (Figure 3(b)). HPV16 controls phosphorylation cascade so that cell behaviors including cell proliferation and differentiation, cell survival, and apoptosis are broken.

**Figure 6:**
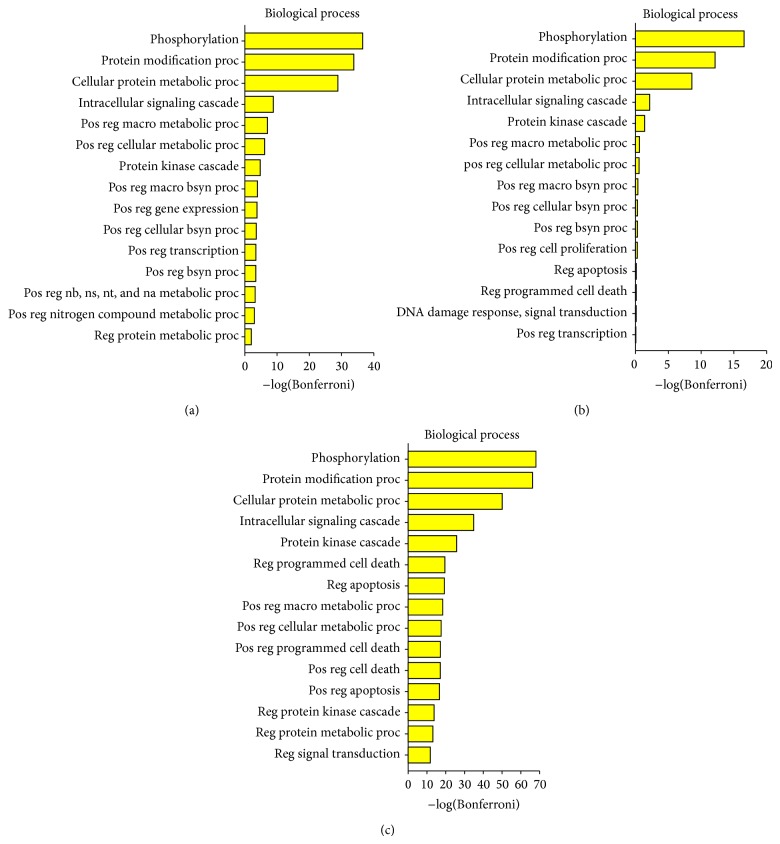
Significantly enriched GO biological process terms. (a) Enriched results of E1, E2, L2, and L1. (b) Enriched results of E4. (c) Enriched results of E5, E6, and E7. Here only fifteen most significant terms are shown. Bonferroni collected *P* values were transformed by −log⁡_10_⁡.

Five proteins (E1, E2 (and E4), L1, and L2) are encoded by all known PVs. There is a hypothesis that the ancestral papillomavirus did not contain adaptive proteins and only need the core set to meet the basic requirements of a viral infection [11]. In the process of evolution, HPV16 produced all of the adaptive proteins. It was surprising that the top four of biological process enrichment of all adaptive proteins were the same as core set's top four, and then processes involving apoptosis and death were enriched for core set (Figures 6(a) and 6(c)). This showed that HPV16 would evolve carcinogenicity, but only on the condition that its own reproduction had been ensured. The E4 protein has the functions of adaptive proteins and core set (Figure 6) but prefers the latter. In other words, as a part of the proteins encoded by all known PVs E4 must first guarantee viral reproduction and then together with adaptive proteins enhance the carcinogenicity of HPV16.

## 4. Conclusions

Significant challenges currently impair experiments to get a more complete map of interactions between HPV16 and human proteins, facilitating computational methods to detect potential interactions. Sequence features are popular because of its simplicity and availability. SVM has been shown to perform well in multiple areas including detecting remote protein homologies, evaluating microarray expression data, and checking new interactions [33, 58, 59]. On the basis of facts above we predicted new interactions between HPV16 and human proteins. The predicted set and other known interactions were integrated and filtered, providing a total of 877 interactions between 8 virus and 603 human proteins. According to the interactions between the virus and human proteins, we plotted the distribution of targeted host proteins. The distribution showed that the virus enlarged its scope of influence by interacting with host proteins as much as possible. HPV16 alters a broad array of cell cycle progression by a number of PPIs. Utilizing integrated human PPI network the enrichment of targeted host proteins as a function of their degree or betweenness was calculated. Results suggested that HPV16 was highly prone to interact with hub proteins and bottleneck proteins, perhaps because these proteins control critical processes in the human cell [17]. PPC was used as a measure of diversity. In the light of their distributions, targeted human proteins effectively mediated the diversity of influenced signaling pathways which helps virus mediate the infection. GO term enrichment was utilized to perform the comprehensive functional analysis. We found that cell behaviors of host cell were broken; the HPV16 produced many other functions by evolution, but it was based on the premise that its own reproduction has been guaranteed.

The integration and analysis of virus-host interactions boosts our knowledge about the function of HPV16 proteins and relations between virus and human proteins. These results improve our understanding of HPV16 pathogenesis and provide potential new targets for interfering with either HPV16 or human at key points in the infection. Our results may point to important areas of research to guide further experimental studies.

## Supplementary Material

The Supplementary Material provides three tables which describe positive training set, positive test set and all set, respectively. The Table S1 lists the positive training set which contains 174 interactions. The Table S2 lists the positive test set based on literature curation with indication of Pubmed ID. Column 1, virus protein; column 2, targeted human protein/gene description; column3, Pubmed ID supported this interaction. The Table S3 lists the all set which consists of filtering results of predicted set, positive test set and training set.

## References

[1] Crow J. M. (2012). HPV: the global burden. *Nature*.

[2] Arbyn M., Castellsagué X., de sanjosé S. (2011). Worldwide burden of cervical cancer in 2008. *Annals of Oncology*.

[3] Bosch F. X., Broker T. R., Forman D. (2013). Comprehensive control of human papillomavirus infections and related diseases. *Vaccine*.

[4] Tornesello M. L., Buonaguro L., Giorgi-Rossi P., Buonaguro F. M. (2013). Viral and cellular biomarkers in the diagnosis of cervical intraepithelial neoplasia and cancer. *BioMed Research International*.

[5] Bernard H.-U., Burk R. D., Chen Z., van Doorslaer K., Hausen H. Z., de Villiers E.-M. (2010). Classification of papillomaviruses (PVs) based on 189 PV types and proposal of taxonomic amendments. *Virology*.

[6] Bzhalava D., Guan P., Franceschi S., Dillner J., Clifford G. (2013). A systematic review of the prevalence of mucosal and cutaneous human papillomavirus types. *Virology*.

[7] Schiffman M., Clifford G., Buonaguro F. M. (2009). Classification of weakly carcinogenic human papillomavirus types: addressing the limits of epidemiology at the borderline. *Infectious Agents and Cancer*.

[8] Humans IWGotEoCRt (2012). Biological agents. Volume 100 B. A review of human carcinogens. *IARC monographs on the evaluation of carcinogenic risks to humans/World Health Organization, International Agency for Research on Cancer. Part B*.

[9] Hansen C. N., Nielsen L., Norrild B. (2010). Activities of E7 promoters in the human papillomavirus type 16 genome during cell differentiation. *Virus Research*.

[10] Wang J. W., Roden R. B. S. (2013). L2, the minor capsid protein of papillomavirus. *Virology*.

[11] van Doorslaer K. (2013). Evolution of the Papillomaviridae. *Virology*.

[12] Vande Pol S. B., Klingelhutz A. J. (2013). Papillomavirus E6 oncoproteins. *Virology*.

[13] Wuchty S. (2011). Computational prediction of Host-Parasite protein interactions between *P. falciparum* and *H. sapiens*. *PLoS ONE*.

[14] Yang H., Ke Y., Wang J. (2011). Insight into bacterial virulence mechanisms against host immune response via the *Yersinia pestis*-human protein-protein interaction network. *Infection and Immunity*.

[15] Calderwood M. A., Venkatesan K., Xing L. (2007). Epstein-Barr virus and virus human protein interaction maps. *Proceedings of the National Academy of Sciences of the United States of America*.

[16] de Chassey B., Navratil V., Tafforeau L. (2008). Hepatitis C virus infection protein network. *Molecular Systems Biology*.

[17] Dyer M. D., Murali T. M., Sobral B. W. (2008). The landscape of human proteins interacting with viruses and other pathogens. *PLoS Pathogens*.

[18] Wodak S. J., Méndez R. (2004). Prediction of protein-protein interactions: the CAPRI experiment, its evaluation and implications. *Current Opinion in Structural Biology*.

[19] Cui G., Fang C., Han K. (2012). Prediction of protein-protein interactions between viruses and human by an SVM model. *BMC Bioinformatics*.

[20] Ewing R. M., Chu P., Elisma F. (2007). Large-scale mapping of human protein-protein interactions by mass spectrometry. *Molecular Systems Biology*.

[21] Rual J.-F., Venkatesan K., Hao T. (2005). Towards a proteome-scale map of the human protein-protein interaction network. *Nature*.

[22] Stelzl U., Worm U., Lalowski M. (2005). A human protein-protein interaction network: a resource for annotating the proteome. *Cell*.

[23] Croft D., O'Kelly G., Wu G. (2011). Reactome: a database of reactions, pathways and biological processes. *Nucleic Acids Research*.

[24] Licata L., Briganti L., Peluso D. (2012). MINT, the molecular interaction database: 2012 update. *Nucleic Acids Research*.

[25] Kerrien S., Aranda B., Breuza L. (2012). The IntAct molecular interaction database in 2012. *Nucleic Acids Research*.

[26] Prasad T. S. K., Goel R., Kandasamy K. (2009). Human protein reference database—2009 update. *Nucleic Acids Research*.

[27] Schaefer C. F., Anthony K., Krupa S. (2009). PID: the pathway interaction database. *Nucleic Acids Research*.

[28] Orchard S., Ammari M., Aranda B. (2014). The MIntAct project—intAct as a common curation platform for 11 molecular interaction databases. *Nucleic Acids Research*.

[29] Prieto C., de Las Rivas J. (2006). APID: agile protein interaction DataAnalyzer. *Nucleic Acids Research*.

[30] Navratil V., de chassey B., Meyniel L. (2009). VirHostNet: a knowledge base for the management and the analysis of proteome-wide virus-host interaction networks. *Nucleic Acids Research*.

[31] Mei S. (2013). Probability weighted ensemble transfer learning for predicting interactions between HIV-1 and human proteins. *PLoS ONE*.

[32] Guo Y., Yu L., Wen Z., Li M. (2008). Using support vector machine combined with auto covariance to predict protein-protein interactions from protein sequences. *Nucleic Acids Research*.

[33] Shen J., Zhang J., Luo X. (2007). Predicting protein-protein interactions based only on sequences information. *Proceedings of the National Academy of Sciences of the United States of America*.

[34] Chang C.-C., Lin C.-J. (2011). LIBSVM: a Library for support vector machines. *ACM Transactions on Intelligent Systems and Technology*.

[35] Lukesova E., Boucek J., Rotnaglova E. (2014). High level of tregs is a positive prognostic marker in patients with HPV-positive oral and oropharyngeal squamous cell carcinomas. *BioMed Research International*.

[36] Wuchty S., Siwo G., Ferdig M. T. (2010). Viral organization of human proteins. *PLoS ONE*.

[37] Ashburner M., Ball C. A., Blake J. A. (2000). Gene ontology: tool for the unification of biology. *Nature Genetics*.

[38] Dennis G., Sherman B. T., Hosack D. A. (2003). DAVID: database for annotation, visualization, and integrated discovery. *Genome Biology*.

[39] Huang D. W., Sherman B. T., Lempicki R. A. (2009). Systematic and integrative analysis of large gene lists using DAVID bioinformatics resources. *Nature Protocols*.

[40] Doolittle J. M., Gomez S. M. (2011). Mapping protein interactions between dengue virus and its human and insect hosts. *PLoS Neglected Tropical Diseases*.

[41] Doolittle J. M., Gomez S. M. (2010). Structural similarity-based predictions of protein interactions between HIV-1 and Homo sapiens. *Virology Journal*.

[42] Habig M., Smola H., Dole V. S., Derynck R., Pfister H., Smola-Hess S. (2006). E7 proteins from high- and low-risk human papillomaviruses bind to TGF-*β*-regulated Smad proteins and inhibit their transcriptional activity. *Archives of Virology*.

[43] Larochelle S., Pandur J., Fisher R. P., Salz H. K., Suter B. (1998). Cdk7 is essential for mitosis and for in vivo Cdk-activating kinase activity. *Genes and Development*.

[44] Schachter M. M., Fisher R. P. (2013). The CDK-activating kinase Cdk7: taking yes for an answer. *Cell Cycle*.

[45] Bisteau X., Paternot S., Colleoni B. (2013). CDK4 T172 phosphorylation is central in a CDK7-dependent bidirectional CDK4/CDK2 interplay mediated by p21 phosphorylation at the restriction point. *PLoS Genetics*.

[46] Schachter M. M., Merrick K. A., Larochelle S. (2013). A Cdk7-Cdk4 T-loop phosphorylation cascade promotes G1 progression. *Molecular Cell*.

[47] Harper J. W., Elledge S. J. (1998). The role of Cdk7 in CAK function, a retro-retrospective. *Genes & Development*.

[48] Fisher R. P. (2005). Secrets of a double agent: CDK7 in cell-cycle control and transcription. *Journal of Cell Science*.

[49] Holcakova J., Muller P., Tomasec P. (2014). Inhibition of post-transcriptional RNA processing by CDK inhibitors and its implication in anti-viral therapy. *PLoS ONE*.

[50] Bergvall M., Melendy T., Archambault J. (2013). The E1 proteins. *Virology*.

[51] Doorbar J. (2013). The E4 protein; structure, function and patterns of expression. *Virology*.

[52] Connell L. E., Helfman D. M. (2006). Myosin light chain kinase plays a role in the regulation of epithelial cell survival. *Journal of Cell Science*.

[53] Shen M.-R., Furla P., Chou C.-Y., Ellory C. J. (2002). Myosin light chain kinase modulates hypotonicity-induced Ca^2+^ entry and Cl- channel activity in human cervical cancer cells. *Pflugers Archiv European Journal of Physiology*.

[54] Shin D. H., Chun Y.-S., Lee K.-H., Shin H.-W., Park J.-W. (2009). Arrest defective-1 controls tumor cell behavior by acetylating myosin light chain kinase. *PLoS ONE*.

[55] Kaczkowski B., Rossing M., Andersen D. K. (2012). Integrative analyses reveal novel strategies in HPV11,−16 and −45 early infection. *Scientific Reports*.

[56] Pérez-Plasencia C., Vázquez-Ortiz G., López-Romero R., Piña-Sanchez P., Moreno J., Salcedo M. (2007). Genome wide expression analysis in HPV16 cervical cancer: identification of altered metabolic pathways. *Infectious Agents and Cancer*.

[57] Huang D. W., Sherman B. T., Lempicki R. A. (2009). Bioinformatics enrichment tools: Paths toward the comprehensive functional analysis of large gene lists. *Nucleic Acids Research*.

[58] Jaakkola T., Diekhans M., Haussler D. (1999). Using the Fisher kernel method to detect remote protein homologies. *Proceedings of the 7th International Conference on Intelligent Systems for Molecular Biology (ISMB ’99)*.

[59] Brown M. P. S., Grundy W. N., Lin D. (2000). Knowledge-based analysis of microarray gene expression data by using support vector machines. *Proceedings of the National Academy of Sciences of the United States of America*.

