# Supplementary material for: Improving the Understanding of Pathogenesis of Human Papillomavirus 16 via Mapping Protein-Protein Interaction Network
Source: Biomed Res Int. 2015 Apr 15;2015:890381. doi: 10.1155/2015/890381 (PMC4414230; doi:10.1155/2015/890381)
Supplement: Supplementary file 1 — The Supplementary Material provides three tables which describe positive training set, positive test set and all set, respectively. The Table S1 lists the positive training set which contains 174 interactions. The Table S2 lists the positive test set based on literature curation with indication of Pubmed ID. Column 1, virus protein; column 2, targeted human protein/gene description; column3, Pubmed ID supported this interaction. The Table S3 lists the all set which consists of filtering results of predicted set, positive test set and training set. [file 890381.f1.pdf]

Table S1. List of the positive training set

HPV16	HUMAN PROTEIN	HPV16	HUMAN PROTEIN
L1	O00410	E7	O00716
L1	P11142	E7	O15304
L1	P52292	E7	O43586
L1	Q14974	E7	O96020
L1	Q92973	E7	P01100
L2	O00410	E7	P01106
L2	O00754	E7	P05412
L2	P48029	E7	P06400
L2	P52292	E7	P06493
L2	Q14974	E7	P07437
L2	Q15831	E7	P07900
L2	Q8TAE8	E7	P08246
L2	Q92973	E7	P08686
L2	Q92993	E7	P10914
L2	Q96EK4	E7	P14618
L2	Q99471	E7	P17174
L2	Q9GZM7	E7	P17275
L2	Q9HBE1	E7	P17535
L2	Q9Y679	E7	P17936
E1	P63279	E7	P20226
E1	Q12959	E7	P20248
E1	Q15645	E7	P21980
E2	O14617	E7	P22392
E2	O60885	E7	P24864
E2	P04637	E7	P24941
E2	P11387	E7	P28749
E2	P17676	E7	P30153
E2	P20226	E7	P30512
E2	P21675	E7	P30532
E2	P22736	E7	P30740
E2	P31274	E7	P36955
E2	P49715	E7	P38398
E2	P54368	E7	P38936
E2	P56537	E7	P46527
E2	P63244	E7	P49411
E2	P82094	E7	P51784
E2	Q00403	E7	P62191
E2	Q15637	E7	P62826
E2	Q16637	E7	P62851
E2	Q8TD31	E7	P63279

HPV16	HUMAN PROTEIN	HPV16	HUMAN PROTEIN
E2	Q92547	E7	P67775
E2	Q92831	E7	P68032
E2	Q99966	E7	P68400
E2	Q9BX70	E7	P78396
E2	Q9H0C5	E7	P98082
E2	Q9Y244	E7	Q00978
E2	Q9Y570	E7	Q01094
E6	O00220	E7	Q01860
E6	O14907	E7	Q08050
E6	O15350	E7	Q08999
E6	O43166	E7	Q09472
E6	P01106	E7	Q13309
E6	P04198	E7	Q13426
E6	P04637	E7	Q13478
E6	P10914	E7	Q13547
E6	P18887	E7	Q13573
E6	P19438	E7	Q13616
E6	P23142	E7	Q14186
E6	P26045	E7	Q14188
E6	P33993	E7	Q14209
E6	P38398	E7	Q14839
E6	P49023	E7	Q14978
E6	P49815	E7	Q16254
E6	P52292	E7	Q4G0J3
E6	P78352	E7	Q5T4S7
E6	Q05086	E7	Q7L099
E6	Q09472	E7	Q92769
E6	Q12959	E7	Q92793
E6	Q12986	E7	Q92831
E6	Q13158	E7	Q96CA5
E6	Q13227	E7	Q96EY1
E6	Q13478	E7	Q96RI1
E6	Q14160	E7	Q96TC7
E6	Q14257	E7	Q9BZX2
E6	Q14653	E7	Q9NVX7
E6	Q14974	E7	Q9Y3A3
E6	Q16512	E7	Q9Y3D9
E6	Q16611	E7	Q9Y3Z3
E6	Q92793	E4	Q96SB4
E6	Q92973	E5	O00410
E6	Q96JN8	E5	P00533
E6	Q96QZ7	E5	P04626

HPV16	HUMAN PROTEIN	HPV16	HUMAN PROTEIN
E6	Q9HD26	E5	P07333
E6	Q9P0J0	E5	P09619
E6	Q9UHV2	E5	P30443
E7	O00268	E5	P30455
E7	O00602	E5	P51572

Table S2. List of the positive test set

HPV16	Targeted protein/gene description	PMID
E1	p70	9696837
E1	TRIP13	9223484
E2	Androgenreceptor	17092759
E2	Cell division cycle protein 20 homolog	16222116
E2	CASP8 and FADD-like apoptosis regulator	20882347
E2	Cleavage and polyadenylation specificity factor subunit4	22617423
E2	Probable ATP-dependent RNA helicase DDX11	17189189
E2	E1A-binding protein p400	20133580
E2	Fizzy-related protein homolog	16222116
E2	Glutamate receptor-interacting protein 1	17376404
E2	Kinesin-like protein KIF20A	23341853<br>22761572<br>17135315
E2	Importin subunit alpha-3	20193720
E2	Importin subunit alpha-6	20193720
E2	E3 ubiquitin-protein ligase Mdm2	19004934
E2	Nuclear receptor-interacting protein 1	22177699
E2	hSNF5	20829602
E2	Serine/arginine-rich splicing factor 1	19187948
E2	Serine/arginine-rich splicing factor 5	19187948
E2	Tax1-binding protein 1	19109394
E2	UBC9	18619639
E2	Sp1	8083979
E2	P300	10982355
E4	keratin18	14694114
E4	Cdk1	12208959
E4	CyclinB	
E4	Cdk2	16540140
E4	CyclinA	
E4	p42 MAPK	19211765
E4	TopBP1	17287259
E5	Endothelin-1 receptor	23731971
E5	Calnexin	
E5	c-jun	
E5	c-fos	
E5	ErbB4	
E5	Phospholipase C-γ1	
E5	p27	
E5	p21	
E5	Bax	

HPV16	Targeted protein/gene description	PMID
E5	Bcl-2	23731971
E5	Fas	
E5	Connexin 43	
E5	16Kvacuolar-ATPasesubunit	
E5	c-Cbl	
E5	ERK1	
E5	ERK2	
E5	CD1d	
E5	Cyclo-oxygenase-2	
E5	ABCC1	22810586
E5	AMFR	
E5	APOB	
E5	ATAD3A	
E5	ATP1A1	
E5	ATP2A2	
E5	ATP2B4	
E5	ATP5J	
E5	ATP5O	
E5	ATP6	
E5	C20orf3	
E5	CAV1	
E5	CD44	
E5	CHRM2	
E5	COPA	
E5	COX2	
E5	CPT1A	
E5	CSPG4	
E5	DAD1	
E5	DDOST	
E5	EMD	
E5	F2R	
E5	FAF2	
E5	GPAA1	
E5	HM13	
E5	IFITM3	
E5	IGF2R	
E5	ITGB1	
E5	MAVS	
E5	MBOAT7	
E5	MLEC	
E5	MMP14	

HPV16	Targeted protein/gene description	PMID
E5	NDUFA4	22810586
E5	PDIA3	
E5	PHB2	
E5	PIGK	
E5	PIGS	
E5	PIGT	
E5	PIGU	
E5	PKD2	
E5	PLD3	
E5	PON2	
E5	PRAF2	
E5	PTPLAD1	
E5	PTPN1	
E5	PTRF	
E5	QPCTL	
E5	RAB18	
E5	RABAC1	
E5	REEP5	
E5	RPL13A	
E5	RPL21	
E5	RPN2	
E5	RTN4	
E5	S100A13	
E5	SEC11A	
E5	SEC61B	
E5	SEC62	
E5	SERPINE2	
E5	SLC1A5	
E5	SLC2A1	
E5	SLC3A2	
E5	SPNS1	
E5	SQRDL	
E5	SRPRB	
E5	SSR3	
E5	SSR4	
E5	STOM	
E5	STT3A	
E5	SURF4	
E5	TAP1	
E5	TAPBP	
E5	TMEM33	

HPV16	Targeted protein/gene description	PMID
E5	TMEM66	22810586
E5	TMPO	
E5	TRAM1	
E5	TRPV4	
E5	VKORC1	
E6	TADA3	12235159
E6	USP15	23015706
E6	CYLD	18977328
E6	BARD1	17678435
E6	TIP-2	15767424
E6	GADD34/PP1	15060162
E6	MUPPI	11000240
E6	PKA C-alpha	11127819
E6	ATF3	20167600
E6	PKA C-beta	11127819
E6	PML-IV	15107834
E6	TYK2	10523853
E6	UBC9	21510985
E6	SMAD3	17020941
E6	Procaspase 8	17267478
E6	Zyxin	11689660
E6	PKN	10809724
E6	MAGI-2	11571640
E6	CARM1	21963854
E6	PIASy	16793547
E6	PATJ	17287269
E6	MAML1	22249263
E6	PRMT1	21963854
E6	HIPK2	22110707
E6	TERT	19843693
E6	HERC2	19553310
E6	MAGI-3	15378012
E6	PKN	10809724
E6	SET7	21963854
E6	COL6A2	22810586
E6	PDZRN3	
E6	PSMA1	
E6	PSMA4	
E6	PSMA6	
E6	PSMB1	
E6	PSMB2	

HPV16	Targeted protein/gene description	PMID
E6	PSMC1	22810586
E6	PSMC2	
E6	PSMC3	
E6	PSMC4	
E6	PSMC5	
E6	PSMC6	
E6	PSMD1	
E6	PSMD11	
E6	PSMD12	
E6	PSMD13	
E6	PSMD14	
E6	PSMD3	
E6	PSMD4	
E6	PSMD7	
E6	PSMD8	
E6	RPL13A	
E6	SRRM2	
E6	UBR5	
E7	CAPN1	17977825
E7	CENPC	22890155
E7	CHUK	11986318
E7	CUL2	17609271
E7	CUL3	22232672
E7	DYRK1A	18468476
E7	FHL2	17093190
E7	HIF1A	21148070
E7	NCOA1	16775354
E7	NUMA1	19052088
E7	PML	15657429
E7	SMAD1	16710631<br>12145312
E7	SMAD2	
E7	SMAD3	
E7	SMAD4	
E7	SMARCA4	12372840
E7	TAF1C	7478615
E7	TAP1	11580231
E7	ZBTB17	22099967
E7	KDM1A	15016876
E7	IKAPPAB KINASE COMPLEX	11986318
E7	ATPase of the 26 S proteasome	9374493
E7	TBG1	17913829

HPV16	Targeted protein/gene description	PMID
E7	E2F6	18579589
E7	GSTP1	19826491
E7	NME1	17103045
E7	HTRA1	21148070
E7	AES	22810586
E7	AP2A1	
E7	AP2M1	
E7	ARHGAP35	
E7	BEND7	
E7	BYSL	
E7	CCND3	
E7	CDK4	
E7	CDK5	
E7	CLK2	
E7	CNP	
E7	E2F5	
E7	FAM164C	
E7	JAK1	
E7	KIAA1377	
E7	LMO2	
E7	PCNA	
E7	PIN4	
E7	POLR1C	
E7	PRKAA2	
E7	PSMC2	
E7	PTPN14	
E7	PTPN21	
E7	REL	
E7	RPL9	
E7	SQRDL	
E7	SRP19	
E7	SRRM2	
E7	SYT16	
E7	TCEB1	
E7	TCEB2	
E7	TEAD4	
E7	TXNDC5	
E7	ZBTB25	
E7	ZGPAT	
E7	ZNF408	
E7	ZNF417	

HPV16	Targeted protein/gene description	PMID
L1	CyPB	19629175
L1	Syndecan-1	19878308<br>23601855
L1	Syndecan-2	
L1	HSPG	
L2	CyPB	19629175
L2	SNX17	22151726
L2	HSC70	15140951
L2	DYNLT1	21166973
L2	DYNLT3	
L2	FURIN	24816794

Table S3. List of the all set

HPV16	HUMAN PROTEIN	HPV16	HUMAN PROTEIN
L1	O95757	E7	P08134
L1	P08107	E7	P08235
L1	P11142	E7	P08238
L1	P23284	E7	P08246
L1	P34741	E7	P08572
L1	P52292	E7	P09211
L1	P52294	E7	P09543
L1	Q14974	E7	P10275
L1	Q92598	E7	P10301
L2	O00141	E7	P10827
L2	O00505	E7	P10828
L2	O00754	E7	P10914
L2	O14757	E7	P11473
L2	O43318	E7	P11802
L2	O75914	E7	P12004
L2	O75962	E7	P12931
L2	O95251	E7	P13747
L2	P00519	E7	P13762
L2	P05129	E7	P14618
L2	P05771	E7	P14625
L2	P06493	E7	P14859
L2	P07711	E7	P15153
L2	P07858	E7	P15336
L2	P09958	E7	P15531
L2	P10074	E7	P16591
L2	P10398	E7	P17275
L2	P11142	E7	P17535
L2	P11802	E7	P17612
L2	P15622	E7	P17936
L2	P17612	E7	P19525
L2	P21127	E7	P19784
L2	P22694	E7	P19793
L2	P23284	E7	P20226
L2	P23458	E7	P20337
L2	P24941	E7	P21127
L2	P25774	E7	P21675
L2	P27361	E7	P21980
L2	P28482	E7	P22392
L2	P31751	E7	P22694
L2	P41743	E7	P22736

HPV16	HUMAN PROTEIN	HPV16	HUMAN PROTEIN
L2	P42684	E7	P23258
L2	P45983	E7	P23458
L2	P45984	E7	P24593
L2	P48029	E7	P24821
L2	P49137	E7	P24941
L2	P49840	E7	P25098
L2	P49841	E7	P27361
L2	P50613	E7	P28482
L2	P51955	E7	P28749
L2	P52292	E7	P29375
L2	P52294	E7	P29590
L2	P53667	E7	P30281
L2	P63172	E7	P30511
L2	Q00535	E7	P30532
L2	Q00536	E7	P30740
L2	Q04759	E7	P31751
L2	Q05655	E7	P32969
L2	Q13164	E7	P34947
L2	Q13177	E7	P35398
L2	Q13188	E7	P35998
L2	Q13464	E7	P36955
L2	Q13976	E7	P37231
L2	Q14164	E7	P38398
L2	Q14974	E7	P38936
L2	Q15746	E7	P39060
L2	Q15759	E7	P41743
L2	Q15831	E7	P42025
L2	Q16566	E7	P42684
L2	Q16659	E7	P45983
L2	Q5S007	E7	P45984
L2	Q5VST9	E7	P46100
L2	Q5VT25	E7	P46459
L2	Q6P0Q8	E7	P46527
L2	Q7KZI7	E7	P46734
L2	Q7L7X3	E7	P48729
L2	Q8TAE8	E7	P49116
L2	Q8TD19	E7	P49137
L2	Q8WYB5	E7	P49411
L2	Q92993	E7	P49759
L2	Q96BR1	E7	P49760
L2	Q96EK4	E7	P49840
L2	Q96GD4	E7	P49841

HPV16	HUMAN PROTEIN	HPV16	HUMAN PROTEIN
L2	Q96J92	E7	P50613
L2	Q96RU7	E7	P50750
L2	Q9BYP7	E7	P51784
L2	Q9GZM7	E7	P51812
L2	Q9H2G2	E7	P51955
L2	Q9H2K8	E7	P52564
L2	Q9HBE1	E7	P53667
L2	Q9HBH9	E7	P55055
L2	Q9HC98	E7	P55072
L2	Q9P286	E7	P56524
L2	Q9UBS0	E7	P60709
L2	Q9UEW8	E7	P60763
L2	Q9UHD2	E7	P60953
L2	Q9UKE5	E7	P61006
L2	Q9Y4K4	E7	P61026
L2	Q9Y5S2	E7	P61106
L2	Q9Y679	E7	P61158
E1	O14745	E7	P61160
E1	O75970	E7	P61163
E1	P62191	E7	P61586
E1	P62333	E7	P62136
E1	P62837	E7	P62191
E1	P63279	E7	P62333
E1	P68036	E7	P62736
E1	P78352	E7	P62826
E1	Q07157	E7	P62834
E1	Q12923	E7	P62837
E1	Q12959	E7	P63000
E1	Q14005	E7	P63261
E1	Q15599	E7	P63279
E1	Q15645	E7	P67775
E1	Q86UL8	E7	P68032
E1	Q96SB3	E7	P68036
E2	O00505	E7	P68104
E2	O14727	E7	P68371
E2	O15164	E7	P68400
E2	O15350	E7	P78396
E2	O15519	E7	P79483
E2	O43172	E7	P84095
E2	O43815	E7	P98082
E2	O60885	E7	Q00534
E2	O60907	E7	Q00535

HPV16	HUMAN PROTEIN	HPV16	HUMAN PROTEIN
E2	O95235	E7	Q00978
E2	P04150	E7	Q01094
E2	P04637	E7	Q01860
E2	P06401	E7	Q02750
E2	P08047	E7	Q03468
E2	P08235	E7	Q03518
E2	P09017	E7	Q04759
E2	P10275	E7	Q04864
E2	P10276	E7	Q05513
E2	P10827	E7	Q05655
E2	P10828	E7	Q07869
E2	P11387	E7	Q08050
E2	P17676	E7	Q08117
E2	P19793	E7	Q08209
E2	P20226	E7	Q08881
E2	P21675	E7	Q08999
E2	P22736	E7	Q09472
E2	P37231	E7	Q12778
E2	P43034	E7	Q12830
E2	P49116	E7	Q12873
E2	P53618	E7	Q13105
E2	P53621	E7	Q13153
E2	P54368	E7	Q13164
E2	P56537	E7	Q13177
E2	P62873	E7	Q13188
E2	P62879	E7	Q13217
E2	P63244	E7	Q13285
E2	P63279	E7	Q13309
E2	Q00987	E7	Q13464
E2	Q04724	E7	Q13478
E2	Q07666	E7	Q13489
E2	Q07869	E7	Q13523
E2	Q07955	E7	Q13547
E2	Q09472	E7	Q13554
E2	Q12830	E7	Q13555
E2	Q13243	E7	Q13573
E2	Q13285	E7	Q13618
E2	Q15637	E7	Q13627
E2	Q16637	E7	Q13813
E2	Q8TD31	E7	Q13895
E2	Q92793	E7	Q13976
E2	Q92830	E7	Q14188

HPV16	HUMAN PROTEIN	HPV16	HUMAN PROTEIN
E2	Q92831	E7	Q14192
E2	Q969H0	E7	Q14653
E2	Q96FC9	E7	Q14978
E2	Q96L91	E7	Q14980
E2	Q99697	E7	Q15139
E2	Q9BX70	E7	Q15329
E2	Q9BZK7	E7	Q15370
E2	Q9H0C5	E7	Q15418
E2	Q9UIF8	E7	Q15572
E2	Q9UKB1	E7	Q15645
E2	Q9UM11	E7	Q15669
E2	Q9Y297	E7	Q15746
E2	Q9Y570	E7	Q15788
E6	O00141	E7	Q15796
E6	O00220	E7	Q15797
E6	O00308	E7	Q15811
E6	O00487	E7	Q15831
E6	O14639	E7	Q16254
E6	O14640	E7	Q16520
E6	O14733	E7	Q16539
E6	O14746	E7	Q16644
E6	O14757	E7	Q16659
E6	O14763	E7	Q16665
E6	O14908	E7	Q5S007
E6	O14920	E7	Q5VST9
E6	O14936	E7	Q6P0Q8
E6	O14965	E7	Q71U36
E6	O15111	E7	Q7L099
E6	O15164	E7	Q7L0Q8
E6	O15350	E7	Q7L7X3
E6	O43166	E7	Q8N4C8
E6	O43294	E7	Q8N5A5
E6	O43318	E7	Q8NBS9
E6	O60674	E7	Q8TD19
E6	O60711	E7	Q8WYK2
E6	O75116	E7	Q92743
E6	O75528	E7	Q92753
E6	O75970	E7	Q92769
E6	O95071	E7	Q92793
E6	O95714	E7	Q92830
E6	P00519	E7	Q92831
E6	P01106	E7	Q92985

HPV16	HUMAN PROTEIN	HPV16	HUMAN PROTEIN
E6	P04198	E7	Q93008
E6	P04637	E7	Q93034
E6	P05129	E7	Q96B97
E6	P05771	E7	Q96CA5
E6	P07948	E7	Q96EY1
E6	P08138	E7	Q96GD4
E6	P10914	E7	Q96L33
E6	P12110	E7	Q96RG2
E6	P14778	E7	Q96T23
E6	P16591	E7	Q96TC7
E6	P17612	E7	Q9BX66
E6	P18887	E7	Q9BXR5
E6	P19438	E7	Q9BY41
E6	P19525	E7	Q9BZ95
E6	P21127	E7	Q9BZL6
E6	P21675	E7	Q9BZX2
E6	P21810	E7	Q9H2K8
E6	P22607	E7	Q9H2X6
E6	P22694	E7	Q9HAZ1
E6	P23142	E7	Q9HBH0
E6	P24941	E7	Q9HBH9
E6	P25098	E7	Q9HC98
E6	P25205	E7	Q9NRH3
E6	P25786	E7	Q9NRY4
E6	P26045	E7	Q9P289
E6	P29074	E7	Q9P2H0
E6	P29350	E7	Q9UBN7
E6	P29590	E7	Q9UIF8
E6	P29597	E7	Q9UJX6
E6	P31751	E7	Q9UK32
E6	P33993	E7	Q9UKE5
E6	P34947	E7	Q9UKV0
E6	P35241	E7	Q9ULU4
E6	P35555	E7	Q9UMD9
E6	P35998	E7	Q9UN37
E6	P36507	E7	Q9UQM7
E6	P38398	E7	Q9Y237
E6	P41240	E7	Q9Y3Z3
E6	P41743	E7	Q9Y4E8
E6	P42680	E7	Q9Y5K6
E6	P46531	E7	Q9Y6N5
E6	P46934	E4	P05783

HPV16	HUMAN PROTEIN	HPV16	HUMAN PROTEIN
E6	P46937	E4	P06493
E6	P49023	E4	P11802
E6	P49137	E4	P24941
E6	P49721	E4	P27361
E6	P49736	E4	P28482
E6	P49815	E4	P45983
E6	P50613	E4	P49759
E6	P51451	E4	P49760
E6	P51665	E4	P50613
E6	P51888	E4	P78362
E6	P51955	E4	Q00534
E6	P52292	E4	Q00535
E6	P52294	E4	Q13523
E6	P52943	E4	Q15746
E6	P53667	E4	Q16539
E6	P60900	E4	Q96SB4
E6	P62191	E4	Q9H2X6
E6	P62333	E4	Q9HAZ1
E6	P63279	E5	O00141
E6	P78352	E5	O00483
E6	P78504	E5	O14733
E6	P98095	E5	O14757
E6	Q00978	E5	O14787
E6	Q02750	E5	O14920
E6	Q02763	E5	O14965
E6	Q02779	E5	O15111
E6	Q04721	E5	O15260
E6	Q04759	E5	O15533
E6	Q05086	E5	O43292
E6	Q05209	E5	O43318
E6	Q05513	E5	O43353
E6	Q05655	E5	O60674
E6	Q06124	E5	O95819
E6	Q07157	E5	P00403
E6	Q07812	E5	P00519
E6	Q07817	E5	P00533
E6	Q07820	E5	P01100
E6	Q09013	E5	P01889
E6	Q09472	E5	P01891
E6	Q12805	E5	P01903
E6	Q12830	E5	P01911
E6	Q12923	E5	P01912

HPV16	HUMAN PROTEIN	HPV16	HUMAN PROTEIN
E6	Q12959	E5	P04440
E6	Q13153	E5	P04626
E6	Q13158	E5	P04844
E6	Q13177	E5	P05023
E6	Q13188	E5	P05129
E6	Q13227	E5	P05412
E6	Q13424	E5	P05556
E6	Q13464	E5	P05771
E6	Q13478	E5	P06213
E6	Q13554	E5	P06239
E6	Q13555	E5	P06241
E6	Q13642	E5	P06493
E6	Q13976	E5	P07093
E6	Q14005	E5	P07333
E6	Q14160	E5	P07947
E6	Q14192	E5	P07948
E6	Q14257	E5	P07949
E6	Q14653	E5	P08172
E6	Q14790	E5	P08195
E6	Q14974	E5	P09619
E6	Q15139	E5	P09769
E6	Q15349	E5	P11166
E6	Q15418	E5	P11717
E6	Q15599	E5	P11802
E6	Q15746	E5	P12931
E6	Q15831	E5	P13762
E6	Q15942	E5	P16070
E6	Q16512	E5	P16234
E6	Q16611	E5	P16591
E6	Q16644	E5	P16615
E6	Q5S007	E5	P17302
E6	Q5TCQ9	E5	P17612
E6	Q5VT25	E5	P18031
E6	Q684P5	E5	P19174
E6	Q6P0Q8	E5	P19525
E6	Q7L7X3	E5	P20036
E6	Q86UL8	E5	P21860
E6	Q8IX03	E5	P22607
E6	Q8N2W9	E5	P22681
E6	Q8N4C8	E5	P22694
E6	Q8TBB1	E5	P23458
E6	Q8TD19	E5	P23634

HPV16	HUMAN PROTEIN	HPV16	HUMAN PROTEIN
E6	Q8WTS6	E5	P24941
E6	Q92585	E5	P25116
E6	Q92793	E5	P27105
E6	Q92796	E5	P27361
E6	Q92985	E5	P27449
E6	Q96GD4	E5	P27824
E6	Q96J02	E5	P28482
E6	Q96JN8	E5	P29317
E6	Q96PU5	E5	P29323
E6	Q96QZ7	E5	P30101
E6	Q96RG2	E5	P30511
E6	Q96SB3	E5	P30530
E6	Q99435	E5	P35354
E6	Q99460	E5	P35968
E6	Q99466	E5	P36507
E6	Q99873	E5	P38936
E6	Q9BXR5	E5	P39656
E6	Q9BZL6	E5	P41240
E6	Q9H0M0	E5	P41743
E6	Q9H2K8	E5	P42166
E6	Q9H2X6	E5	P42167
E6	Q9H3D4	E5	P42679
E6	Q9H4G0	E5	P42680
E6	Q9HBX9	E5	P43403
E6	Q9HBY8	E5	P43405
E6	Q9HC98	E5	P46527
E6	Q9HD26	E5	P46778
E6	Q9NQC7	E5	P46977
E6	Q9NRM7	E5	P48047
E6	Q9NYK1	E5	P49841
E6	Q9P0J0	E5	P50402
E6	Q9P289	E5	P50416
E6	Q9UBN6	E5	P50613
E6	Q9UDY2	E5	P51451
E6	Q9UHV2	E5	P51571
E6	Q9UIF8	E5	P51572
E6	Q9UJA3	E5	P51955
E6	Q9UKE5	E5	P53621
E6	Q9ULJ8	E5	P53667
E6	Q9UNM6	E5	P57088
E6	Q9UPQ7	E5	P61803
E6	Q9UQM7	E5	P79483

HPV16	HUMAN PROTEIN	HPV16	HUMAN PROTEIN
E6	Q9Y2C9	E5	Q00535
E6	Q9Y2J2	E5	Q00765
E6	Q9Y2R2	E5	Q02750
E6	Q9Y2U5	E5	Q02779
E6	Q9Y4E8	E5	Q03518
E6	Q9Y6R4	E5	Q04759
E7	O00141	E5	Q04912
E7	O00602	E5	Q05397
E7	O14646	E5	Q05513
E7	O14733	E5	Q05655
E7	O14757	E5	Q06187
E7	O14829	E5	Q07812
E7	O14920	E5	Q08345
E7	O14936	E5	Q13153
E7	O14965	E5	Q13164
E7	O15111	E5	Q13177
E7	O15164	E5	Q13418
E7	O15304	E5	Q13563
E7	O15379	E5	Q13976
E7	O43318	E5	Q14289
E7	O43524	E5	Q15139
E7	O43586	E5	Q15165
E7	O43918	E5	Q15303
E7	O60229	E5	Q15418
E7	O60264	E5	Q15746
E7	O60341	E5	Q16288
E7	O60674	E5	Q16539
E7	O75116	E5	Q16620
E7	O75461	E5	Q16659
E7	O75962	E5	Q29980
E7	O95163	E5	Q5S007
E7	O95782	E5	Q6P0Q8
E7	O95819	E5	Q7L7X3
E7	O96017	E5	Q7Z434
E7	O96020	E5	Q8IV08
E7	O96028	E5	Q8N4C8
E7	P00505	E5	Q8TD19
E7	P00519	E5	Q96CS3
E7	P00749	E5	Q96J92
E7	P01019	E5	Q99442
E7	P01100	E5	Q99584
E7	P01106	E5	Q99623

HPV16	HUMAN PROTEIN	HPV16	HUMAN PROTEIN
E7	P01111	E5	Q9BYP7
E7	P01891	E5	Q9BZL6
E7	P01903	E5	Q9H2K8
E7	P01911	E5	Q9H2V7
E7	P01912	E5	Q9H490
E7	P02675	E5	Q9HBA0
E7	P04150	E5	Q9HC98
E7	P04350	E5	Q9HDC9
E7	P04440	E5	Q9NP72
E7	P05129	E5	Q9NQC3
E7	P05412	E5	Q9P289
E7	P05771	E5	Q9UI14
E7	P06239	E5	Q9UKE5
E7	P06241	E5	Q9UM73
E7	P06401	E5	Q9UNL2
E7	P06493	E5	Q9Y2U5
E7	P07384	E5	Q9Y572
E7	P07437	E5	Q9Y5M8
E7	P07900	E5	Q9Y6N5
E7	P07947	E5	Q9Y6R4
E7	P07948		

Matlab source code

```
%%%%%%%%%%%%%%%%%%%%%%%%%%%%%%%%%%%%%%%%%%%%%%%
%These codes have been tested in matlab2011a(win7 OS).
% The former three functions were named as key functions to calculated enrichment
% and PPC. The fourth function was used to map ROC curve and obtain AUC and
%AUPR. The other functions were called by key functions and didn't need the readers
% to call.
%%%%%%%%%%%%%%%%%%%%%%%%%%%%%%%%%%%%%%%%%%%%%%%
function [re_human,save_human,PPC]=Runmap_PPC(pathway_filename,…
inter_filename,map_human_id)
% Runmap_PPC.m              This code is used to calculate Pathway Participation
%                           Coefficient(PPC) of targeted human proteins
% Input:
%   pathway_filename        File containing 224 different pathways which was
%                           downloaded from PID.
%   inter_filename          File containing non-redundant interaction pairs of
%                           human proteins.
%   map_human_id            The human proteins of calculating PPC
%Output:
%   re_human                The human protein which PPC cannot be calculated
%   save_human              The human protein which PPC can be calculated
%   PPC                     PPC of save_human
```

```
[corr_human,all_pathway]=getid_pathway(pathway_filename);
[left_inter,right_inter]=textread(inter_filename,'%s %s');
sub_id=intersect(map_human_id,corr_human);
[re_human,save_human]=getyong(sub_id,map_human_id);
uniq_human=unique(save_human);
onebound=size(uniq_human,1);
new_left_inter=[left_inter;right_inter];new_right_inter=[right_inter;left_inter];
[new_left_inter,vsort]=sort(new_left_inter);new_right_inter=new_right_inter(vsort,:);
[allhuman_id,startend]=getindex(new_left_inter);
%[all_pathway,vsort]=sort(all_pathway);corr_human=corr_huma%n(vsort,:);
part_PPC=zeros(onebound,1);
for ii=1:onebound
    look_human=uniq_human(ii);
    v=strcmp(look_human,allhuman_id);vindex=find(v==1);
    subindex=(startend(vindex,1):startend(vindex,2))';
    sub_protein=new_right_inter(subindex,:);
    [part_pathway,~]=getpathway(all_pathway,corr_human,sub_protein);
    [look_pathway,~]=getpathway(all_pathway,corr_human,look_human);
    [~,save_pathway]=getyong(look_pathway,part_pathway);
    part_PPC(ii,1)=getPPC(save_pathway);
end
PPC=DYCmap(save_human,uniq_human,part_PPC);
```

```
end
%%%%%%%%%%%%%%%%%%%%%%%%%%%%%%%%%%%%%%%%%%%%%%
function all_PPC=Runrandom_PPC(pathway_filename,inter_filename, …
map_human_id,count)
%Runrandom_PPC.m           This code is used to calculate Pathway Participation
%                          Coefficient (PPC) of random human proteins
%Input:
%   The former three variables are similar to Runmap_PPC.m
%   count                  Repetition times
%Output:
% all_PPC                  PPC matrix
[corr_human,all_pathway]=getid_pathway(pathway_filename);
A=unique(corr_human);B=numel(A);
[left_inter,right_inter]=textread(inter_filename,'%s %s');
sub_id=intersect(map_human_id,corr_human);
[~,save_human]=getyong(sub_id,map_human_id);
uniq_human=unique(save_human);onebound=numel(uniq_human);
new_left_inter=[left_inter;right_inter];new_right_inter=[right_inter;left_inter];
[new_left_inter,vsort]=sort(new_left_inter);new_right_inter=new_right_inter(vsort,:);
[allhuman_id,startend]=getindex(new_left_inter);
all_PPC=zeros(size(save_human,1),count);
```

```
for jj=1:count
    random_count=randperm(B);random_count=random_count';
    part_random=random_count(1:onebound,:);part_human=A(part_random,:);
    part_PPC=zeros(onebound,1);
    for ii=1:onebound
        look_human=part_human(ii);
        v=strcmp(look_human,allhuman_id);vindex=find(v==1);
        subindex=(startend(vindex,1):startend(vindex,2))';
        sub_protein=new_right_inter(subindex,:);
        [part_pathway,~]=getpathway(all_pathway,corr_human,sub_protein);
        [look_pathway,~]=getpathway(all_pathway,corr_human,look_human);
        [~,save_pathway]=getyong(look_pathway,part_pathway);
        part_PPC(ii,1)=getPPC(save_pathway);
    end
    part_all_human=DYCmap(save_human,uniq_human,part_human);
    all_PPC(:,jj)=DYCmap(part_all_human,part_human,part_PPC);
end
end
%%%%%%%%%%%%%%%%%%%%%%%%%%%%%%%%%%%%%%%%%%%%%
function [value_split,Etk,fisher]=Runall_enrichment(humanid,protein_id,degree,…
max_value,next_step,count)
% Runall_enrichment.m          This code is used to calculate repeatedly the
```

```
%                                  enrichment of targeted human proteins
%Input:
%    humanid                       The human proteins of calculating enrichment
%    protein_id                    The all human protein in the integrated PPI network
%    degree                        The degree (betweenness) of protein_id
%    max_value                     The maximum of degree (betweenness)
%    next_step                     The count of logarithmic segmentation
%    count                         Repetition times
% Output:
%    value_split                   The number of logarithmic segmentation
%    Etk                           The enrichment of humanid
%    fisher                        The P value of fisher's exact test
all_Ntk=zeros(next_step,count);
all_Nrtk=zeros(next_step,count);all_fisher=zeros(next_step,count);
for ii=1:count
    [value_split,Ntk,Nrtk]=Runmap_enrichment(humanid,protein_id,degree,…
    max_value,next_step);
    fisher_p=getfisher(Ntk,Nrtk);
    all_fisher(:,ii)=fisher_p;
    all_Ntk(:,ii)=Ntk;
    all_Nrtk(:,ii)=Nrtk;
end
```

```
all_Etk=all_Ntk./all_Nrtk;
Etk=mean(all_Etk,2);
fisher=mean(all_fisher,2);
end
%%%%%%%%%%%%%%%%%%%%%%%%%%%%%%%%%%%%%%%%%%%%%
function [AUC,AUPR,Acc]=ROCcompute(predictList,trueList,plotOption,…
split_value)
%ROCcompute.m          This code is used to map ROC curve and obtain AUC
]%                     and AUPR.
%Input:
%   predictList        Prediction score matrix or vector
%   trueList           True labels
%   plotOption         A switch whether ploting ROC or not, if plotOption=1,
%                      ploting ROC;
%Output:
%   AUC                The AUC value
%   AUPR               The AUPR value
%   Acc                The accuracy vector
if nargin==3
    split_value=size(predictList,1);
end
if size(predictList,2)>1
```

```
        predictList=reshape(predictList,numel(predictList),1);
        trueList=reshape(trueList,numel(trueList),1);
end

low=min(predictList);
high=max(predictList);
threshold=linspace(high,low,split_value);

Sen=zeros(1,split_value); Spe=zeros(1,split_value);
Pre=zeros(1,split_value);dottoline=0;
Acc=zeros(1,split_value);
for I=1:split_value
        Vector=zeros(numel(predictList),1);
        v=predictList>=threshold(I);Vector(v)=1;

        tp=sum(Vector==1&trueList==1);
        tn=sum(Vector==0&trueList==0);
        np=sum(Vector==1&trueList==0);
        nn=sum(Vector==0&trueList==1);

        Sen(I)=tp/(tp+nn);
        Spe(I)=tn/(tn+np);
```

```
        if tp+np==0
            Pre(I)=1;
        else
            Pre(I)=tp/(tp+np);
        end
        Acc(I)=(tn+tp)/(tn+tp+np+nn);
end

Sen=[0,Sen];Spe=[1,Spe];Pre=[1,Pre];
Acc=[sum(trueList==0)/length(trueList) Acc];

AUC=abs(trapz(1-Spe,Sen));
AUPR=abs(trapz(Sen,Pre));

if nargin==2
        plotOption=1;
end

if plotOption==1
        plot(1-Spe,Sen,'-','Color',[0.7176,0.5686,0.5647],'LineWidth',2.5);
        axis([-0.01 1.00 0 1.01]);
        xlabel('false positive rate');
```

```
        ylabel('true positive rate');


        figure;
        plot(Sen,Pre,'-','Color',[0.7176,0.5686,0.5647],'LineWidth',2.5);
        axis([0 1.01 0 1.01]);
        xlabel('Sen');
        ylabel('Pre');
end
%%%%%%%%%%%%%%%%%%%%%%%%%%%%%%%%%%%%%%%%
function [headers,remains]=getid_pathway(filename)


alllines=textread(filename,'%[^\n]');
onebound=numel(alllines);
headers=cell(onebound,1);remains=cell(onebound,1);
for ii=1:onebound
        headers{ii}=alllines{ii}(1:6);
        remains{ii}=alllines{ii}(end-5:end);
end
end
%%%%%%%%%%%%%%%%%%%%%%%%%%%%%%%%%%%%%%%%
function [re_human,save_human]=getyong(sub_id,map_human_id)
re_human=setdiff(map_human_id,sub_id);save_human={};
```

```
onebound=size(sub_id,1);
for ii=1:onebound
    v=strcmp(sub_id(ii),map_human_id);
    vindex= v==1;
    save_human=[save_human;map_human_id(vindex,:)];
end
end
%%%%%%%%%%%%%%%%%%%%%%%%%%%%%%%%%%%%%%%%%%%%%%
function [part_pathway,part_human]=getpathway(old_pathway,old_human,…
look_human)
onebound=size(look_human,1);
part_pathway={};part_human={};

for ii=1:onebound
    v=strcmp(look_human{ii},old_human);
    vindex=find(v==1);
    part_pathway=[part_pathway;old_pathway(vindex,:)];
    part_human=[part_human;old_human(vindex,:)];
end
end
%%%%%%%%%%%%%%%%%%%%%%%%%%%%%%%%%%%%%%%%%%%%%%
function part_PPC=getPPC(part_pathway)
```

```
[~,count]=getcount(part_pathway);
square_count=sum(count.^2);
sum_count=(sum(count)).^2;
part_PPC=square_count./sum_count;
end
%%%%%%%%%%%%%%%%%%%%%%%%%%%%%%%%%%%%%%%%%%%%%%%
function fisher_p=getfisher(Ntk,Nrtk)
all_num=Ntk(1);
onebound=size(Ntk,1);
fisher_p=zeros(onebound,1);

for ii=1:onebound
    target_high_hub=Ntk(ii);non_high_hub=Nrtk(ii);
    fourth_table=[target_high_hub,all_num-target_high_hub;non_high_hub,…
    all_num-non_high_hub];
    p=FisherExtest[1](fourth_table);
    fisher_p(ii)=p;
end
end
%%%%%%%%%%%%%%%%%%%%%%%%%%%%%%%%%%%%%%%%%%%%%%%
function [uniqueid,startend]=getindex(oldcolumn)
oldcolumn=sort(oldcolumn);
```

```
[uniqueid,index]=unique(oldcolumn);
vLen=index-[0;index(1:end-1,1)];
onebound=size(vLen,1);
startend=zeros(onebound,2);L=1;
for ii=1:onebound
    startend(ii,1)=L;
    startend(ii,2)=L+vLen(ii)-1;
    L=L+vLen(ii);
end
end
%%%%%%%%%%%%%%%%%%%%%%%%%%%%%%%%%%%%%%%%%%%%%%%%%%%%%%
function [subid,count]=getcount(oldid)
sortid=sort(oldid);
[subid,index]=unique(sortid);
count=index-[0;index(1:end-1,1)];
end
%%%%%%%%%%%%%%%%%%%%%%%%%%%%%%%%%%%%%%%%%%%%%%%%%%%%%%
function list2=DYCmap(list,origin,newcolumn)
[list,vSort]=sort(list);
[~,vRev]=sort(vSort);

[list,vEnd]=unique(list);
```

```
vLen=vEnd-[0;vEnd(1:end-1)];


[~,~,id]=intersect(list,origin);

V=zeros(sum(vLen),1);L=1;

for I=1:size(id,1)

        v=L:L+vLen(I)-1;

        V(v)=id(I);

        L=L+vLen(I);

end

V=V(vRev);


list2=newcolumn(V);

end
```


1. Thorvaldsen S, Flå T, Willassen NP: **DeltaProt: a software toolbox for comparative genomics**. *BMC Bioinform* 2010, **11**(1):573.
